# A New Shut-Off1Read at the Surgical Section, Buffalo Academy of Medicine, May, 1899.

**Published:** 1899-09

**Authors:** J. Henry Dowd

**Affiliations:** Buffalo, N. Y., Genito-urinary surgeon, Buffalo Hospital Sisters of Charity


### New Instrument.

### A NEW SHUT-OFF.[1]

By J. HENRY DOWD, M. D., Buffalo, N. Y.,
Genito-urinary surgeon, Buffalo Hospital Sisters of Charity.

THE contrivance I exhibit you is a simple affair, but one I trust will fill a much felt want. Its simplicity of handling must appeal to all, for with one hand and slight pressure of the thumb a stream of water can be directed moderately or with force on any wound or operating field. Furthermore, by simply releasing the thumb complete closure takes place. Its advantages as compared with others of this sort may be summed up as follows: No fear of rust or corrosion from fluids, as with Esmarch's, where leakage cannot but occur

*We have diligently though unsuccessfully tried to obtain this cut from the Jeffrey-Fell Company, Ellicott Square, Buffalo, N. Y.*—EDITOR.

around the metal fittings, thus in a short time causing them to corrode and break.

The operator or his assistant, with either hand, has complete control, only having to drop or release his thumb to stop the flow. This one point should appeal to all surgeons who daily see the nozzle

1. Read at the Surgical Section, Buffalo Academy of Medicine, May, 1899.

of a douche-bag put into the mouth of the latter when they want irrigation stopped. The cheapness is an important factor, the makers being able to furnish them at about half the price of similar shut-offs. Although I have had one of these in use for six months or more, with constant pressure on the rubber tubing, there is not the least sign of cutting of the latter at site of contact. For those who do not use an irrigator daily and do not want the tubing compressed continuously I have affixed a small catch which can be used to relieve pressure when the douche is not in use.

288 FRANKLIN STREET.

